# A Randomized Clinical Trial Testing Hydroxychloroquine for Reduction of SARS-CoV-2 Viral Shedding and Hospitalization in Early Outpatient COVID-19 Infection

**DOI:** 10.1128/spectrum.04674-22

**Published:** 2023-03-02

**Authors:** Adam M. Spivak, Bradley J. Barney, Tom Greene, Richard Holubkov, Cody S. Olsen, Jordan Bridges, Raj Srivastava, Brandon Webb, Frances Sebahar, Ainsley Huffman, Christina F. Pacchia, J. Michael Dean, Rachel Hess

**Affiliations:** a Department of Medicine, University of Utah, Salt Lake City, Utah, USA; b Department of Pediatrics, University of Utah, Salt Lake City, Utah, USA; c Department of Population Health Sciences, University of Utah, Salt Lake City, Utah, USA; d Senior Medical Executive Director, Intermountain Healthcare Delivery Institute, Intermountain Healthcare, Salt Lake City, Utah, USA; e Division of Infectious Diseases, Intermountain Healthcare, Salt Lake City, Utah, USA; f Utah Clinical and Translational Science Institute, University of Utah, Salt Lake City, Utah, USA; University of Wisconsin-Madison

**Keywords:** COVID-19, hydroxychloroquine, SARS-CoV-2

## Abstract

Early in the COVID-19 pandemic, no effective treatment existed to prevent clinical worsening of COVID-19 among recently diagnosed outpatients. At the University of Utah, Salt Lake City, Utah, we conducted a phase 2 prospective parallel group randomized placebo-controlled trial (NCT04342169) to determine whether hydroxychloroquine given early in disease reduces the duration of SARS-CoV-2 shedding. We enrolled nonhospitalized adults (≥18 years of age) with a recent positive diagnostic test for SARS-CoV-2 (within 72 h of enrollment) and adult household contacts. Participants received either 400 mg hydroxychloroquine by mouth twice daily on day 1 followed by 200 mg by mouth twice daily on days 2 to 5 or oral placebo with the same schedule. We performed SARS-CoV-2 nucleic acid amplification testing (NAAT) on oropharyngeal swabs on days 1 to 14 and 28 and monitored clinical symptomatology, rates of hospitalization, and viral acquisition by adult household contacts. We identified no overall differences in the duration of oropharyngeal carriage of SARS-CoV-2 (hazard ratio of viral shedding time comparing hydroxychloroquine to placebo, 1.21; 95% confidence interval [CI], 0.91, 1.62). Overall, 28-day hospitalization incidence was similar between treatments (4.6% hydroxychloroquine versus 2.7% placebo). No differences were seen in symptom duration, severity, or viral acquisition in household contacts between treatment groups. The study did not reach the prespecified enrollment target, which was likely influenced by a steep decline in COVID-19 incidence corresponding to the initial vaccine rollout in the spring of 2021. Oropharyngeal swabs were self-collected, which may introduce variability in these results. Placebo treatments were not identical to hydroxychloroquine treatments (capsules versus tablets) which may have led to inadvertent participant unblinding. In this group of community adults early in the COVID-19 pandemic, hydroxychloroquine did not significantly alter the natural history of early COVID-19 disease. (This study has been registered at ClinicalTrials.gov under registration no. NCT04342169).

**IMPORTANCE** Early in the COVID-19 pandemic, no effective treatment existed to prevent clinical worsening of COVID-19 among recently diagnosed outpatients. Hydroxychloroquine received attention as a possible early treatment; however, quality prospective studies were lacking. We conducted a clinical trial to test the ability of hydroxychloroquine to prevent clinical worsening of COVID-19.

## INTRODUCTION

COVID-19 is a pandemic illness caused by the novel coronavirus SARS-CoV-2 that has a high mortality among hospitalized patients, despite a benign course in most infected individuals (1, 2). Severe SARS-CoV-2 infection causes acute and potentially lethal respiratory failure that has repeatedly threatened to overwhelm health care systems across the globe due to dramatic surges in hospitalized and critically ill patients (3). Patients hospitalized with COVID-19 typically have been symptomatic for 5 to 7 days prior to admission (4), indicating that there is a window during which an effective intervention might alter the course of illness, lessen disease spread, and alleviate the stress on hospital resources (5).

Given the individual and public health importance of identifying a well-tolerated, easily accessible therapy for early, outpatient COVID-19, in March 2020, we sought to test the safety and efficacy of hydroxychloroquine (HCQ) with respect to reducing viral shedding, clinical symptoms, hospitalization, and household viral acquisition. In cell models, chloroquine both interferes with terminal glycosylation of the ACE2 receptor (the cell surface receptor by which SARS-CoV-2 enters human cells) and increases endosomal pH, which interferes with a crucial step in viral replication (6, 7). HCQ is more potent than chloroquine in a Vero kidney cell model of SARS-CoV-2 infection (8). In independent experiments in this same cell model, chloroquine has shown *in vitro* activity against SARS-CoV-2 (9). Additionally, HCQ has demonstrated *in vitro* efficacy against SARS-CoV (10).

At the time we designed this trial (late March 2020), observational studies and several under powered clinical trials provided conflicting results regarding the efficacy of HCQ in the management of COVID-19. Promising mechanistic and *in vitro* results for HCQ, inconclusive *in vivo* evidence of efficacy, a rapidly worsening pandemic without any known effective treatment, and strong public interest in this therapy at local, national, and global levels all prompted us to initiate a clinical trial to test HCQ *in vivo*. We designed a phase 2, prospective, placebo-controlled, parallel group randomized trial to test our primary hypothesis that HCQ is effective at reducing the duration of viral shedding in outpatients with confirmed COVID-19 when administered within 72 h of diagnosis. We included several secondary endpoints in this trial, including the effect of HCQ on COVID-19 clinical symptoms, hospitalization within 14 days of enrollment, persistence of viral shedding on day 28, and SARS-CoV-2 acquisition among adult household contacts of study participants.

## RESULTS

**FIG 1:**
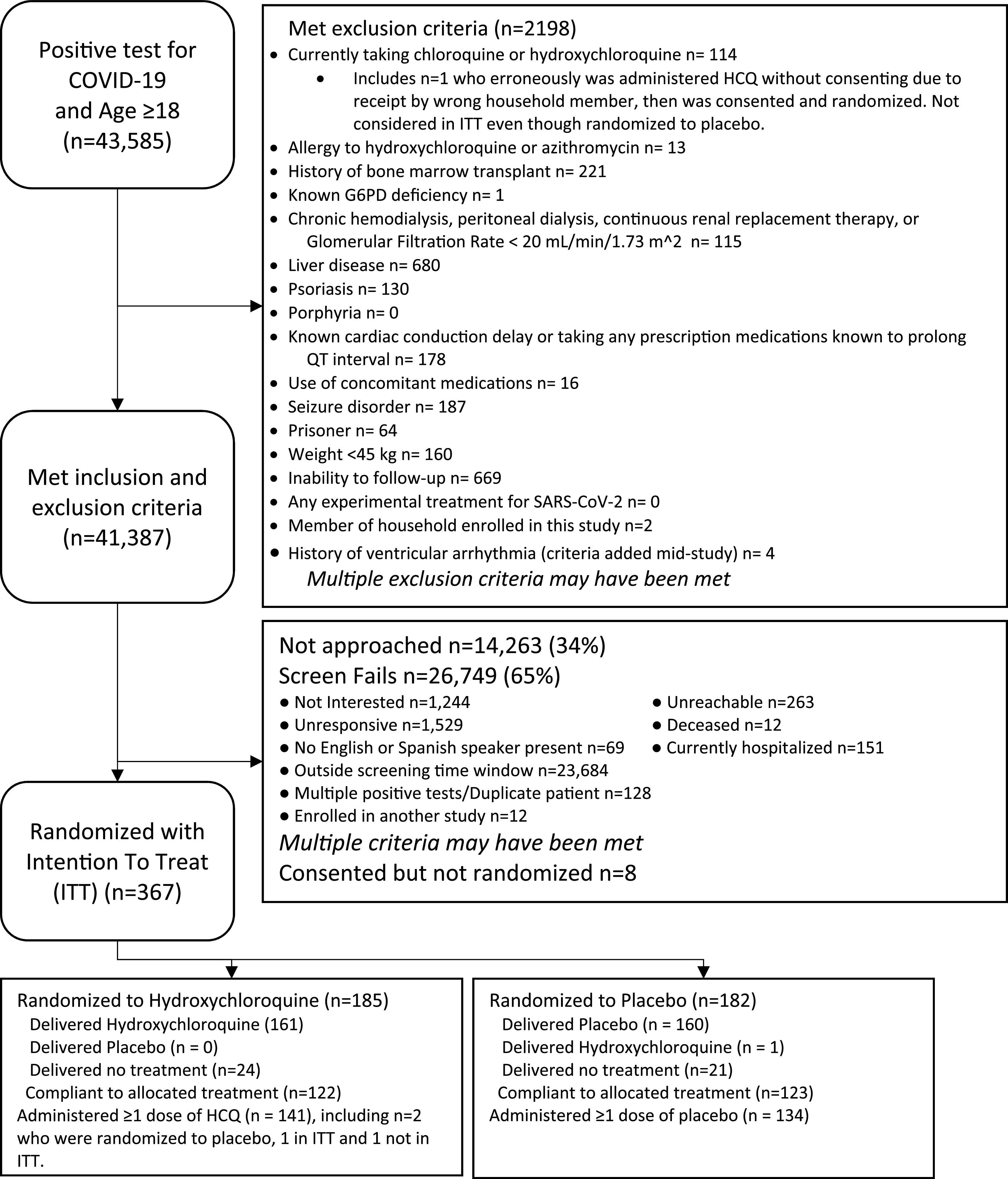
Enrollment and randomization of study participants.

Over the course of study enrollment which ran from April 2020 to April 2021, 43,585 individuals tested positive for SARS-CoV-2 and 41,387 were eligible based on a partial eligibility assessment. Of those individuals, 14,263 were not able to be approached, 26,749 were screened and failed screening, and 367 were enrolled in intention to treat analysis (ITT) (Fig. 1). Among ITT participants, 185 participants were randomized to receive hydroxychloroquine and 182 to receive the placebo. The mean age of participants was 41.9 years (standard deviation, 14.5 years), and 43% of participants self-identified as Latinx. A total of 48% of participants identified as female and 52% as male. Also, 24.5% pf participants had a comorbidity known to predispose them to hospitalization or severe clinical disease due to SARS-CoV-2 infection. These comorbidities included diabetes mellitus, hypertension, chronic pulmonary disease, immunocompromised status, chronic kidney disease, chronic liver disease, morbid obesity, and chronic neurological disease. None of these demographic or clinical characteristics differed significantly based on treatment assignment. Additional participant characteristics by assigned treatment group are shown in Table 1.

**TABLE 1:** Baseline characteristics of participants by treatment group^*a*^

Parameter	Overall data (*n* = 367)	Data by treatment	
		Hydroxychloroquine (*n* = 185)	Placebo (*n* = 182)
Race			
American Indian or Alaskan Native	1 (0.3)	1 (0.5)	0 (0.0)
Asian	2 (0.5)	0 (0.0)	2 (1.1)
Black or African American	2 (0.5)	0 (0.0)	2 (1.1)
Native Hawaiian or Other Pacific Islander	2 (0.5)	1 (0.5)	1 (0.5)
White	165 (45.0)	85 (45.9)	80 (44.0)
Multiracial	3 (0.8)	2 (1.1)	1 (0.5)
Unknown or not reported	192 (52.3)	96 (51.9)	96 (52.7)
Ethnicity			
Hispanic or Latino	158 (43.1)	79 (42.7)	79 (43.4)
Not Hispanic or Latino	171 (46.6)	84 (45.4)	87 (47.8)
Unknown or not reported	38 (10.4)	22 (11.9)	16 (8.8)
Sex			
Male	191 (52.0)	95 (51.4)	96 (52.7)
Female	176 (48.0)	90 (48.6)	86 (47.3)
Age (yrs) (mean [SD])	41.9 (14.53)	42.1 (14.70)	41.8 (14.40)
Age group			
44 yrs of age or younger	212 (57.8)	106 (57.3)	106 (58.2)
45–59 yrs of age	103 (28.1)	52 (28.1)	51 (28.0)
60–74 yrs of age	49 (13.4)	25 (13.5)	24 (13.2)
75 yrs of age or older	3 (0.8)	2 (1.1)	1 (0.5)
Any comorbidity			
No	277 (75.5)	140 (75.7)	137 (75.3)
Yes	90 (24.5)	45 (24.3)	45 (24.7)
Comorbidity			
Diabetes mellitus	28 (7.6)	15 (8.1)	13 (7.1)
Hypertension	52 (14.2)	28 (15.1)	24 (13.2)
Chronic pulmonary disease	8 (2.2)	4 (2.2)	4 (2.2)
Immunocompromised status	7 (1.9)	2 (1.1)	5 (2.7)
Chronic kidney disease	2 (0.5)	2 (1.1)	0 (0.0)
Chronic liver disease	1 (0.3)	1 (0.5)	0 (0.0)
Morbid obesity	9 (2.5)	4 (2.2)	5 (2.7)
Chronic neurological disease	7 (1.9)	4 (2.2)	3 (1.6)

a All values are *n* (%) unless otherwise indicated.

**FIG 2:**
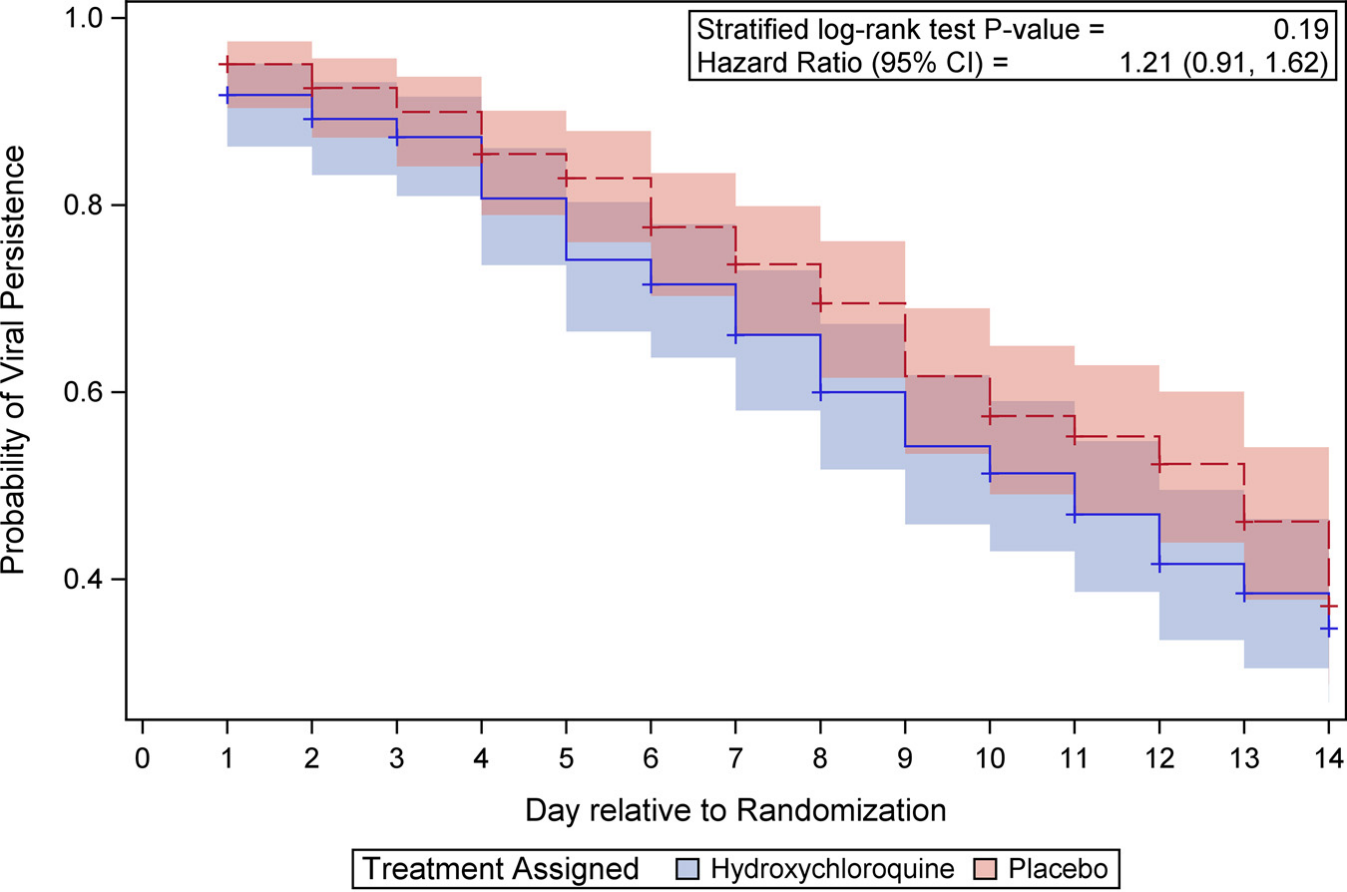
Viral shedding duration during the first 2 weeks following enrollment, based on daily swab test results. Duration was considered to end on the first day of two consecutive negative swabs or on the day of the last available swab result if negative. Participants without a confirmed negative result were considered censored. Kaplan-Meier curves summarize shedding time for the 2 randomized groups. A log-rank test stratified by age groups was performed to compare randomized groups.

Our primary trial outcome was to determine the differences in the kinetics of oropharyngeal clearance of SARS-CoV-2 between those who received hydroxychloroquine and those who received placebo over the 2-week period beginning with randomization. The presence of SARS-CoV-2 was determined using nucleic amplification assays on daily self-collected oropharyngeal swabs on consecutive study days 1 to 14, with day 1 denoting the day study drug was delivered (commonly 1 day after randomization). As shown in Fig. 2, we identified no statistically significant difference in the time to a confirmed negative SARS-CoV-2 test between the two study arms (*P* = 0.19; hazard ratio, 1.21; 95% confidence interval [CI], 0.91, 1.62). An analysis of these data with right-censoring hospitalized patients instead of last value carried forward did not change these results (see Fig. S1 in the supplemental material) (*P* = 0.21; hazard ratio, 1.21; 95% CI, 0.90, 1.62).

In a subgroup analysis by age and symptom duration (greater or less than 5 days of COVID-19-attributable symptoms), the kinetics of SARS-CoV-2 oropharyngeal positivity in each treatment group revealed one statistically significant difference between hydroxychloroquine and placebo—the restricted mean duration for participants 45 to 59 years of age. This finding should be interpreted cautiously given the lack of demonstrated efficacy for the hazard ratio and restricted mean duration overall (Table 2). The intervention was well tolerated (see Table S2 in the supplemental material). Of the 275 participants with reported treatment administration (safety population), 39 (14%) experienced an adverse event (HCQ, *n* = 24 [17%]; placebo, 15 [11%]), 8 (2.9%) experienced a serious adverse event (HCQ, 5 [3.5%]; placebo, 3 [2.2%]), and 5 (1.8%) were hospitalized (28 days) (HCQ, 4 [2.8]; placebo, 1 [0.7%]).

**TABLE 2:** Primary outcome (viral shedding) stratified by age

Parameter	Data by treatment		Difference (95% CI)^*a*^
	Hydroxychloroquine	Placebo	
Restricted mean shedding duration to 14 days			
Overall	9.53	10.40	−0.86 (−1.81, 0.05)
Age ≤44 yrs of age	9.74	10.43	−0.69 (−1.98, 0.61)
Age 45–59 yrs of age	8.51	10.37	−1.86 (−3.95, −0.02)
Age ≥60 yrs of age	10.66	10.31	0.34 (−2.28, 2.91)
Less than 5 days of symptoms^*b*^	10.44	11.29	−0.86 (−2.48, 0.78)
5 or more days of symptoms^*b*^	9.70	9.62	0.07 (−1.40, 1.55)
Median shedding duration^*c*^			
Overall	11	13	−2
Age ≤44 yrs of age	10	13	−3
Age 45–59 yrs of age	9	12	−3
Age ≥60 yrs of age	^*c*^	14	^*c*^
Less than 5 days of symptoms^*b*^	12	14	−2
5 or more days of symptoms^*b*^	11	12	−1

a Negative differences indicate shorter viral shedding durations in the hydroxychloroquine group than those in the placebo group; confidence intervals were estimated from 500 bootstrap samples.

b A total of 47 patients (25, hydroxychloroquine; 22, placebo) were missing symptom onset date and are excluded from this subgroup analysis.

c Confidence intervals were not able to be estimated for median shedding duration; the median and difference were not able to be estimated for the hydroxychloroquine group aged ≥60 years of age.

At day 28, approximately 16% of participants continued to shed virus, although there was no difference in viral persistence between treatment group when stratified by age (*P* = 0.92) (Table 3). A day 28 swab result, without prior evidence of viral shedding cessation based on day 1 to 14 swabs, was missing for 29% of participants (hydroxychloroquine, *n* = 50; placebo, *n* = 58) whose outcomes were imputed.

**TABLE 3:** Persistence of viral shedding on day 28^*a*^

Persistence of viral shedding on day 28	Data (*n* [%]) by treatment		*P* value^*b*^
	Hydroxychloroquine	Placebo	
Overall	30/185 (16.1)	30/182 (16.4)	0.92
Age ≤44 yrs of age	16/106 (14.6)	17/106 (15.6)	
Age 45–59 yrs of age	7/52 (13.8)	8/51 (15.3)	
Age ≥60 yrs of age	7/27 (26.0)	6/25 (22.1)	

a Results from 50 imputations were combined. Outcome was unknown and imputed for 50 hydroxychloroquine and 58 placebo participants. Table counts were rounded after averaging across imputations.

b The *P* value is from a 2-sided Cochran-Mantel-Haenszel test stratified by age category.

**FIG 3:**
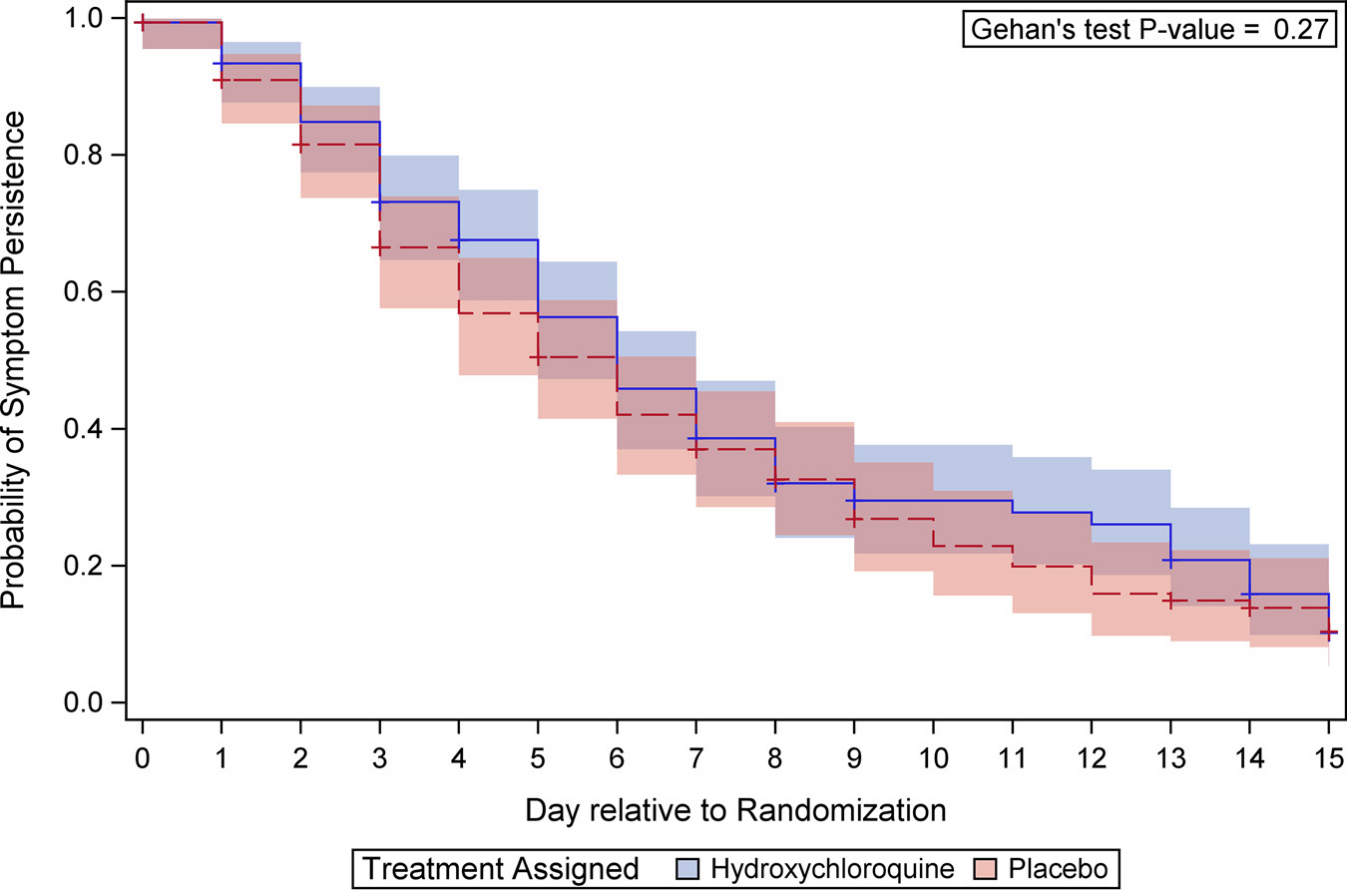
Duration of COVID-19-attributable symptoms evaluated among participants symptomatic at baseline. Duration was defined as the number of days through day 15 that any of the following core symptom(s) exceeded the permissible threshold: not experiencing fever and chills; extremely mild for shortness of breath, diarrhea, and muscle aches; and mild cough and tiredness. Days in hospital were also considered symptomatic. Participants whose last daily assessment was symptomatic were censored. Kaplan-Meier curves summarize symptom duration for the 2 randomized groups. Gehan’s test stratified by age group was performed to compare randomized groups.

Median symptom duration was the same for participants assigned hydroxychloroquine (6 days [interquartile range of 3, 13]) as that for participants assigned placebo (6 days [interquartile range of 3, 10]). By day 15, only an estimated 10% of people remained symptomatic and the duration of COVID-19-attributable symptoms did not differ significantly between treatment groups (*P* = 0.27) (Fig. 3). Average symptom scores also did not differ significantly between groups overall or when stratified by age (Table 4).

**TABLE 4:** Average symptom scores by treatment group

Avg symptom score^*a*^ (*n*, mean [SD]) by treatment	Data (*n*, mean [SD]) by treatment		Unadjusted difference (95% CI)^*b*^	Adjusted difference (95% CI)^*b*^	*P* value^*b*^
	Hydroxychloroquine (*n* = 167)	Placebo (*n* = 165)			
Overall	167, 0.45 (0.63)	165, 0.49 (0.48)	−0.04 (−0.21, 0.12)	−0.10 (−0.26, 0.05)	0.19
Age ≤44 yrs of age	98, 0.38 (0.66)	95, 0.45 (0.37)			
Age 45–59 yrs of age	44, 0.54 (0.50)	47, 0.57 (0.64)			
Age ≥60 yrs of age	25, 0.55 (0.72)	23, 0.50 (0.48)			

a Average symptom score is defined as the 14-day average of daily average symptom scores. Daily averages are the average score on a 5-point scale (0 = not experiencing, 5 = extremely severe) reported from each of the following seven symptoms: fever, chills, cough, tiredness, shortness of breath, muscle aches, and diarrhea. If only one symptom was not documented, the average over the six documented symptoms was used; if more than one item was not documented, that daily average symptom score was considered missing. Missing values were accounted for using last value carried forward for hospitalized participants and multiple imputation for others. Analysis includes participants in the intention-to-treat population with at least one follow-up survey with six or more core symptoms assessed.

b Unadjusted difference and 95% confidence interval (CI) are from simple linear regression; adjusted difference, 95% CI, and *P* value are from a linear regression model testing for a treatment effect with covariates, as follows: baseline symptom score, age group, any comorbidity, age x comorbidity interaction, sex, race, and ethnicity. Results from 50 imputations are combined using standard methods.

As a secondary outcome measure, we evaluated the rate of hospitalization and mortality within 14 days of trial enrollment between arms. No deaths occurred in either trial arm during the course of the trial. A total of 11 participants were hospitalized. Hospitalization rates did not differ significantly based on treatment group (Table 5). The overall stratum-adjusted estimate and 95% confidence interval of the relative risk of hospitalization within 14 days were 1.72 (0.53, 5.62). Given the small counts, the stratum-specific estimates and 95% confidence intervals of relative risks were unwieldy and they are not reported; they were not statistically significant.

**TABLE 5:** Rate of hospitalization by treatment group

Outcome and strata	Data by treatment (*n* [%])		*P* value
	Hydroxychloroquine	Placebo	
Hospitalization within 14 days^*a*^			
ITT^*b*^ population	7/152 (4.6)	4/151 (2.6)	0.36^*c*^
ITT population ≤44 yrs of age	2/87 (2.3)	0/86 (0.0)	
ITT population 45–59 yrs of age	3/42 (7.1)	1/43 (2.3)	
ITT population ≥60 yrs of age	2/23 (8.7)	3/22 (13.6)	
Hospitalization prior to delivery of study drug^*d*^			
ITT population	1/182 (0.5)	1/181 (0.6)	>0.99^*e*^
Hospitalization within 28 days			
ITT population	7/152 (4.6)	4/150 (2.7)	0.541^*e*^
Safety population^*f*^	4/138 (2.9)	1/129 (0.8)	0.372^*e*^

a Hospitalization status unknown for 33 (3 safety) hydroxychloroquine, and 31 (5 safety) placebo participants.

b ITT, intention to treat population.

c The *P* value is from a Cochran-Mantel-Haenszel test stratified by age strata.

d No hospitalizations prior to delivery of study drug were reported in the safety population.

e Each *P* value is from a 2-sided Fisher’s exact test.

f The outcome is summarized by the treatment received; all other summaries show treatment assigned.

As a secondary outcome, we sought to determine whether the rate of SARS-CoV-2 acquisition among adult household members differed between treatment groups. Among the 367 ITT participants, 140 had at least 1 adult household contact positive for SARS-CoV-2 at baseline and 155 had no adult household contact volunteers. A total of 72 participants had at least 1 household contact that provided oropharyngeal swabs for evaluation that were negative at baseline. A total of 49% of adult household contacts acquired COVID-19 during the 14 days of evaluation. No difference was detected between treatment groups (Table 6). Given the nature of these results, missingness in the number of adults in the household, and the limited counts of household acquisitions, we did not fit the planned modified Poisson regression models or stratify by the number of adults in the household.

**TABLE 6:** Adult household contact viral acquisition within 14 days

Strata	Data by treatment^*a*^		Relative risk (95% CI)	*P* value^*b*^
	Hydroxychloroquine	Placebo		
Overall	16/33 (48.5)	19/39 (48.7)	0.96 (0.60, 1.54)	0.87
≤44 yrs of age	7/20 (35.0)	11/24 (45.8)	0.76 (0.36, 1.60)	
45–59 yrs of age	7/9 (77.8)	5/8 (62.5)	1.24 (0.66, 2.36)	
≥60 yrs of age	2/4 (50.0)	3/7 (42.9)	1.17 (0.32, 4.28)	

a No. of households with viral acquisition/no. of eligible households (%). Eligible households are those with at least 2 adults, where no other adult besides the index subject is positive for COVID-19 at baseline, and where another adult submitted swab samples.

b The *P* value is from a 2-sided Cochran-Mantel-Haenszel test stratified by age category.

## DISCUSSION

Early in the COVID-19 pandemic, we rapidly launched one of the first rigorous, placebo-controlled trials investigating the utility of HCQ. This trial provided an important mechanism to inform a critical knowledge gap in a way that safeguarded patient safety. Although several trials have subsequently failed to demonstrate the efficacy of HCQ since the completion of this study, variation in participant enrollment, controls, dosing, and endpoints across trials combined with additional, rigorous data further clarify this point. Our results add compelling evidence that HCQ treatment of early COVID-19 does not result in virologic or symptomatic benefit, prevent progression to severe disease requiring hospitalization, or prevent intrahousehold transmission. The intervention was well-tolerated by participants but demonstrated no statistically significant overall efficacy for any of these outcomes relative to the placebo. Since the completion of our trial, failure to demonstrate HCQ efficacy has been replicated independently by many trial groups worldwide (11–21). *In vivo* measurements of HCQ pharmacokinetics (PK) in one study were discrepant from *in vitro* PK modeling that informed the design of most of these trials (22). Reliance on inadequate *in vitro* HCQ PK modeling may help explain the consistent negative results of this intervention (23).

Our trial did identify several interesting aspects of the natural history of early COVID-19. With regard to the kinetics of viral shedding (measured by the positivity of self-collected oropharyngeal nucleic acid amplification testing for SARS-CoV-2), we observed that participants remained positive for an average of 10 days after study enrollment and that 15% of participants remained positive at day 28. These data are consistent with other studies on viral shedding (24).

We observed a household transmission rate of almost 50% among adult contacts of trial participants. This finding is quite a bit higher than that in other studies (25), perhaps because we measured only transmission in adults, whereas overall detectable household transmission dynamics may be significantly different in children (26). These data must also be interpreted in the context of the transmissibility of the variants circulating at the time of the study, in what was at that time an immune-naive population. Our trial did not meet prespecified enrollment targets due to decreased disease incidence (likely related to vaccine availability in the spring of 2021) and participants not always providing all study swabs. As such, the trial may have been underpowered to detect a significant difference in the primary outcome measure. The initial promise of hydroxychloroquine, based on the well-understood safety profile of the drug *in vivo*, antiviral efficacy observed *in vitro*, and small observational case series early in the pandemic, has not translated into meaningful clinical outcomes in any randomized clinical trial, including the one we describe here.

## MATERIALS AND METHODS

### Design overview.

The Understanding Treatment And Health in the Ongoing coronavirus epidemic (UTAH One trial) was a prospective, placebo-controlled, randomized clinical trial conducted to evaluate the efficacy and safety of HCQ in individuals with confirmed COVID-19 (Clinicaltrials.gov no. NCT04342169). We obtained approval of the University of Utah Institutional Review Board in April 2020 (IRB_00131893) and began enrollment shortly afterward.

### Setting and study population.

Patients undergoing testing for SARS-CoV-2 within the University of Utah Health system were informed of this actively enrolling interventional trial at the time of testing and at the time of positive test. Study personnel were provided with a list of individuals with positive tests, contacted them to verify eligibility, and engaged them in a remote informed consent discussion by telephone, electronic consent, and/or videoconference. The consenting process was performed via a consenting mechanism that confirmed the identity of the patient, provided access to the consent form, and obtained a signature to complete documentation, without requiring physical proximity of research staff and the study participant. The informed consent documentation included Health Insurance Portability and Accountability Act (HIPAA) authorization for access to their medical record. Prior to randomization, study physicians reviewed medications being prescribed for patients on study to confirm eligibility. Study personnel advised patients to contact the study team if any new medications were prescribed during the 5-day treatment course so that the medication could be reviewed for risk of prolonging QT interval (QTc). Full inclusion and exclusion criteria can be found in Table S3 in the supplemental material.

### Randomization.

Individuals were randomized to either the HCQ or placebo arm within 72 h of COVID-19 laboratory-based diagnosis (SARS-CoV-2 nucleic acid amplification test positivity per clinical lab standards). Randomization was stratified by age group and balanced between the two arms using random permuted blocks of size 2 or 4 (block size varies randomly after the initial block). A trial statistician generated the sequence using R version 3.6.0 statistical software.

### Study interventions and procedures.

Participants randomized to the HCQ arm received 400 mg HCQ by mouth twice daily for 1 day and then 200 mg by mouth twice daily for 4 days. This drug dose (2.4 grams over 5 days) falls at the lower end of doses tested in various international trials, but it has proven *in vitro* efficacy in a Vero kidney cell model, with a ratio of >20 between lung tissue trough concentrations and the effective concentration to suppress 50% of viral activity (EC_50_) (8). Participants randomized to the placebo arm received placebo tablets to be taken on the same schedule. Coordinators obtained baseline information at the time of randomization.

Daily assessments (assessments on days 1 through 15) included a review for hospitalization as well as current symptoms. Daily assessments also explicitly solicited the occurrence of rash, symptomatic hypoglycemia, seizure, oxygen supplementation, nausea, vomiting, and impaired vision. Quality-of-life assessments were made at baseline, day 28 ± 2 days, and 6 months ± 2 weeks, using standardized survey instruments, including the EQ-5D-5L, PHQ-9, GAD 7, and PCL 5 (see Table S1 in the supplemental material).

Study nurses fitted with N-100 respirators and appropriate personal protective equipment delivered study medication and taught participants how to perform self-collection of oropharyngeal swabs for SARS-CoV-2 on assessment day 1. Participants then collected swabs on days 1 to 14 and day 28 ± 2 (considering day 1 as day of study drug delivery). Oral swabs were not required for participants hospitalized during the study period, but patients were asked to provide a swab on the day following discharge from the hospital, even if this occurred after day 28. Swabs were refrigerated after collection, placed in an insulated container, and collected by a courier associated with the study three times a week for transport to our central hospital laboratory (ARUP) for testing using a standard clinical SARS-CoV-2 nucleic acid amplification diagnostic assay (the Hologic SARS-CoV-2 qualitative nucleic amplification assay on a Panther System platform). In addition to in-person training on swab collection and storage, written instructions, video, and as-needed discussion with research coordinators were also provided. Household contacts 18 years or older were asked to self-collect oral swabs in the same manner and same daily schedule as the study participant.

Phlebotomy was performed at day 1, day 7 (±2 day), and day 28 (±2 day) in the participant’s home or at a central location as appropriate for a participant’s infectivity. Plasma and peripheral blood mononuclear cells (PBMCs) were processed and stored in the University of Utah Clinical and Translational Science Institute (CTSI) biospecimen repository. Blood samples were used to assess the immune response to SARS-CoV-2, perform a drug screening, investigate viral biology, assess cardiac biomarkers, and develop diagnostic assays. Six months after enrollment, study staff verified subsequent hospitalizations and the vital status of participants.

### Statistical analysis.

We summarized demographic information and comorbidity prevalence overall and by treatment arm. We and tested for baseline differences using Fisher’s exact and Wilcoxon signed-rank tests.

The primary outcome was viral shedding duration during the first 2 weeks after enrollment, based on swab test results. Swabs were considered positive if the oropharyngeal SARS-CoV-2 RNA PCR cycle threshold met the cutoff established as positive for the clinical assay used. Because the protocol stipulated that swabs would not be obtained during hospitalizations and that positive results would be carried forward through hospitalization, swab status was considered positive for all hospital days. Hospitalizations or swabs documented after day 15 were not used for this outcome. Duration was considered to end on the first day of two consecutive negative swabs, allowing days without swab results between two negative swabs or on the last available swab result if negative. Without negative confirmation, the outcome was considered right-censored on the day of their last positive result or day 15. In a sensitivity analysis, we treated hospitalized patients prior to day 15 without a prior confirmed-negative test as right-censored on the day of hospital admission. We fit Kaplan-Meier curves and tested for a difference between treatment arms using a log-rank test stratified by age groups and used the Efron approach to account for ties.

As additional analyses of the primary outcome, we estimated age-stratified differences between treatment groups in 14-day truncated mean and median shedding duration with 95% confidence intervals based on estimates derived from Kaplan-Meier curves. The confidence intervals for each difference were constructed in a manner similar to Calkins et al. (27) for overall and stratified confidence intervals of the restricted mean duration, except that no weights were applied because of our randomized trial design.

For the secondary outcome of the persistence of viral shedding on day 28, we used day 28 swab results if available, day 1 to 14 swab results otherwise if those showed viral shedding cessation, and multiple imputation methods otherwise in order to retain all eligible ITT participants in the analysis. We tested for a treatment effect using a Cochran-Mantel-Haenszel (CMH) test stratified by age group.

The duration of COVID-19-attributable symptoms, an exploratory outcome evaluated among participants symptomatic at baseline, was defined as the number of days through day 15 that any core symptom exceeded the permissible threshold, as follows: not experiencing fever and chills; extremely mild for shortness of breath, diarrhea, and muscle aches; and mild for cough and tiredness. Daily assessments with at least one symptom exceeding a threshold or a hospitalization were considered symptomatic. Otherwise, daily assessments with at least 6 symptoms below the threshold were considered nonsymptomatic. The principal analysis of this outcome required a confirmation of symptom resolution, defined as two consecutive nonsymptomatic assessments, or the last assessment being nonsymptomatic. Assessments with insufficient information were skipped so that only daily assessments with nonmissing outcomes were used to confirm symptom resolution. The confirmation requirement was ignored in a sensitivity analysis. Participants whose last daily assessment was symptomatic were censored. We summarized this outcome by treatment using Kaplan-Meier curves and Gehan’s test stratified by age group.

The secondary outcome of average symptom score was the average level of the 7 aforementioned core symptoms across the 15 daily symptom assessments beginning on the day of study drug delivery, with each ranging from 0 (not experiencing) to 5 (extremely severe). Baseline and subsequent daily averages required at least six core symptoms to be assessed to be considered nonmissing. Although participants with only missing averages were excluded from this analysis, a combination of last value carried forward (for hospitalized days) and multiple imputation (for all others) methods were used so that all participants with at least one originally nonmissing average were included. Average symptom levels were modeled with linear regression adjusted for baseline average symptom level, age group, comorbidity presence, treatment, sex, race, ethnicity, and age-by-comorbidity interaction effects, with interest on the treatment effect.

We compared the proportion of patients hospitalized within 2 weeks (a secondary outcome) by treatment with a two-sided CMH test stratified by age group and estimated relative risks with 95% confidence intervals within each age stratum. We tested for treatment differences in 28-day hospitalization among the ITT and safety (drug-administered per self-report) populations and compared the rate of hospitalization prior to study drug delivery in the two arms using CMH tests.

Finally, we tested for treatment differences in the secondary outcome of adult household contact viral acquisition in the first 2 weeks. This analysis was limited to participants with no adult household contacts positive at baseline, for whom at least one household contact consented and provided swabs. We compared this binary outcome between treatments using a CMH test stratified by age group. Small numbers of eligible household contacts led us to not conduct some additional planned analyses of this cohort.

Analyses were performed in SAS Software (version 9.4; SAS Institute Inc., Cary, NC). Two-sided *P* values less than 0.05 were considered significant. Multiple imputation was performed using chained equations implemented in IVEware (version 0.3; University of Michigan, Ann Arbor, MI), and results from 50 imputations were combined using standard methods (28).
