# Supplementary material for: A Randomized Clinical Trial Testing Hydroxychloroquine for Reduction of SARS-CoV-2 Viral Shedding and Hospitalization in Early Outpatient COVID-19 Infection
Source: Microbiol Spectr. 2023 Mar 2;11(2):e04674-22. doi: 10.1128/spectrum.04674-22 (PMC10101001; doi:10.1128/spectrum.04674-22)
Supplement: Supplemental file 1 — Tables S1 to S3 and Fig. S1. Download spectrum.04674-22-s0001.pdf, PDF file, 0.2 MB [file spectrum.04674-22-s0001.pdf]

Supplementary Tables and Figures

Supplemental Table 1: Schedule of study events

**Study Events**	Day	**0**	**1**	**2**	**3**	**4**	**5**	**6**	**7**	**8**	**9**	**10**	**11**	**12**	**13**	**14**	**28**
			**HCQ or Placebo**														
COVID-19 PCR swab positive		1															
Inclusion / Exclusion criteria		1															
Informed consent discussion / signatures		1	1														
Data collection and adverse event review			1	1	1	1	1	1	1	1	1	1	1	1	1	1	1
Surveys (EQ-5D-5L / PHQ-9 / GAD7 / PLC5)			1														1
HCQ or Placebo administration			1	1	1	1	1										
**Research Specimen Collection**																	
Oropharyngeal self-swab for SARS-CoV-2			1	1	1	1	1	1	1	1	1	1	1	1	1	1	1
Household contact over age 18 oropharyngeal self-swab for SARS-CoV-2			1	1	1	1	1	1	1	1	1	1	1	1	1	1	1
Peripheral blood (40mL, processed and stored in PBMC / plasma biobank)			1						1								1

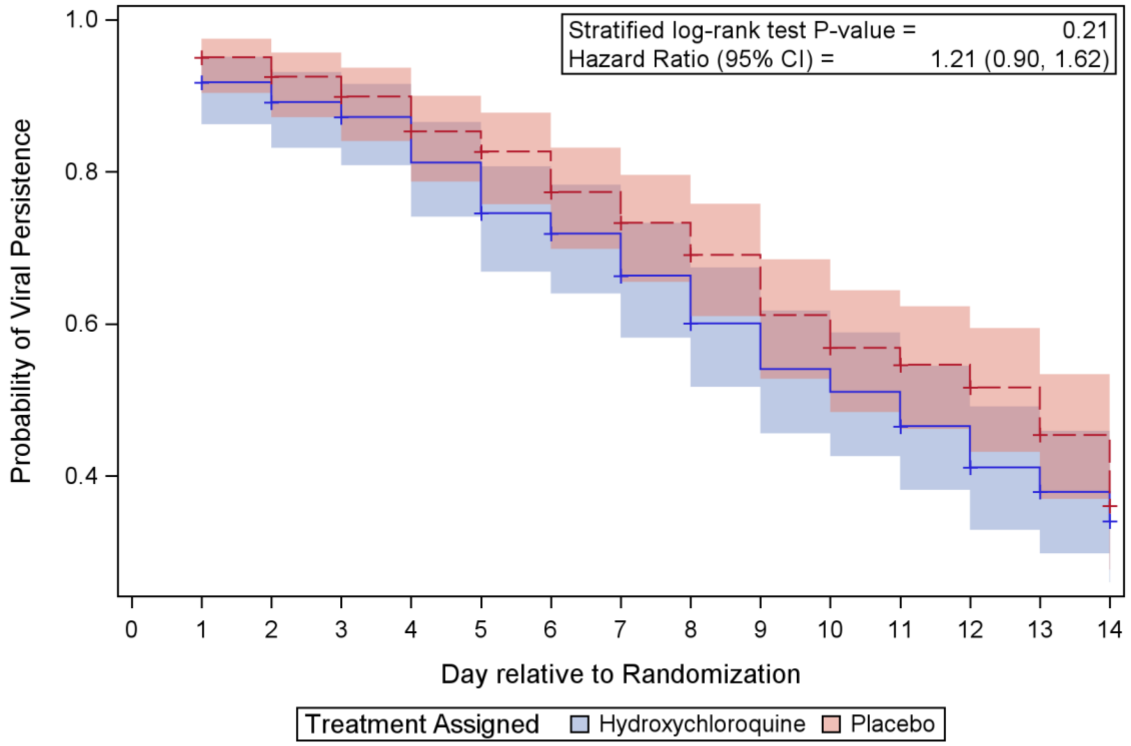

Supplemental Figure 1: Sensitivity analysis of viral shedding duration, where patients hospitalized prior to day 15 without a prior confirmed negative were treated as right censored on the day of hospital admission. For patients not hospitalized prior to day 15, this outcome was unchanged from the one used in the primary analysis. Kaplan-Meier curves summarize shedding time for the 2 randomized treatment groups. A log-rank test stratified by age groups was performed to compare randomized treatment groups.

Supplemental Table 2: Safety outcomes

	Treatment Received			
**Safety Population:** Safety Outcomes	Overall (N = 275)	Hydroxychloroquine (N = 141)	Placebo (N = 134)	P-value
Any Safety Outcome	40 (14.5%)	25 (17.7%)	15 (11.2%)	
Any Adverse Event	39 (14.2%)	24 (17.0%)	15 (11.2%)	0.17
Serious Adverse Event	8 (2.9%)	5 (3.5%)	3 (2.2%)	0.72
Hospitalization within 28 days	5 (1.8%)	4 (2.8%)	1 (0.7%)	0.37

Supplemental Table 3: Trial Inclusion and Exclusion Criteria

Inclusion Criteria for Randomized Group:

- Patient age ≥18 years, competent to provide consent
- Within 72 hours of positive nucleic acid test for SARS-CoV-2 (Initially required within 48 hours, but promptly amended to promote feasibility)

Inclusion Criteria for Household Contact Group:

- Individual age ≥18 living in the same house as someone diagnosed with COVID, competent to provide consent

Exclusion

- Patient already prescribed chloroquine or hydroxychloroquine
- Allergy to hydroxychloroquine
- History of bone marrow or solid organ transplant
- Known G6PD deficiency
- Chronic hemodialysis, peritoneal dialysis, continuous renal replacement therapy or Glomerular Filtration Rate < $20 ml/min/1.73 m^2$
- Known liver disease (e.g. Child Pugh score ≥ B or AST>2 times upper limit)
- Psoriasis
- Porphyria
- Known cardiac conduction delay (QTc > 500mSec)
- Concomitant use of digitalis, flecainide, amiodarone, procainamide, propafenone, or any other prescription medication known to prolong the QT interval
- Seizure disorder
- Prisoner
- Weight < 45kg
- Inability to follow-up – no cell phone or no address or not Spanish or English speaking
- Receipt of any experimental treatment for SARS-CoV-2 (off-label, compassionate use, or trial related) within the 30 days prior to the time of the screening evaluation
- Patient or another member of patient’s household has been already enrolled in this study.
- History of ventricular arrhythmia (exclusion added partway through the enrollment period)
